# Prevalence and risk factors for nausea and vomiting in breast cancer patients undergoing chemotherapy

**DOI:** 10.2340/1651-226X.2026.44933

**Published:** 2026-04-01

**Authors:** Yuhui Feng, Liushan Wei, Qinhong Zou, Xiaoyong Lei, Xiaoyan Yang

**Affiliations:** a School of Pharmaceutical Science, Hengyang Medical College, University of South China, Hengyang, Hunan, People’s Republic of China; b The Second Hospital & Clinical Medical School, Lanzhou University, Lanzhou, Gansu Province, People’s Republic of China; c The College of Pharmacy, Xiangnan University, Chenzhou, Hunan, People’s Republic of China

**Keywords:** Breast cancer, chemotherapy, nausea and vomiting, risk factors, meta-analysis

## Abstract

**Background and purpose:**

Chemotherapy-induced nausea and vomiting (CINV) is a common and severe adverse effect of breast cancer (BC) treatment that compromises treatment adherence and quality of life. This meta-analysis aims to assess the prevalence and risk factors of CINV in BC patients, thereby providing clinical insights for its prevention and improvement.

**Patient/material and methods:**

Relevant literature was identified through an extensive search of electronic databases from their inception up to July 10, 2025: PubMed, Web of Science, Embase, Cochrane, CNKI, Wanfang, and VIP databases on prevalence rates, odds ratios (OR), and corresponding 95% confidence intervals (CI) were extracted for analysis.

**Results:**

The screening process identified 12 eligible studies. The meta-analysis showed that the overall prevalence of CINV in BC patients was 48% (95% CI = 0.37–0.58). Univariate analysis identified the following risk factors: age ≤ 45 years (OR = 3.21, 95% CI = 1.44–7.17) and a history of motion sickness (OR = 4.85, 95% CI = 1.65–14.30). Multivariate analysis showed that age ≤ 45 years (OR = 2.36, 95% CI = 1.78–3.14), history of motion sickness (OR = 2.05, 95% CI = 1.42–2.98), and chemotherapy cycles ≥ 3 (OR = 2.27, 95% CI = 1.28–4.04) were risk factors. In contrast, anxiety (OR = 2.74, 95% CI = 0.66–11.29) and comorbidities (OR = 1.04, 95% CI = 0.72–1.49) were not significantly associated with CINV in BC patients.

**Interpretation:**

This meta-analysis shows that the prevalence of CINV in BC patients is high. It focused on the Asian population and indicated that younger age, history of motion sickness, and more chemotherapy cycles (≥ 3) were risk factors for CINV. Targeting these risk factors may help prevent CINV in BC patients.

## Introduction

Global statistics from the WHO indicate that breast cancer (BC) is the most common cancer among women worldwide [1]. It exhibits high morbidity and mortality, with over 2 million new cases and more than 600,000 deaths annually [1–3]. The pathogenesis of BC is complex, involving genetics, hormone levels, lifestyle, and other factors [4, 5]. Enhancing patient survival and quality of life hinges on early detection and intervention [6]. Significant progress has also been made in the treatment of BC. Patients now benefit from a variety of treatment options, such as surgery, chemotherapy, radiotherapy, endocrine therapy and targeted therapy [7]. Chemotherapy holds an indispensable role in the BC treatment paradigm due to its broad applicability. This is particularly true for triple-negative, HER2-positive, and high-risk hormone receptor-positive BCs, where its efficacy is well-established [8, 9]. Whether it is neoadjuvant chemotherapy to debulk the tumor to facilitate surgery, postoperative chemotherapy to remove micrometastases to minimize the risk of recurrence, or palliative chemotherapy to control the progression of advanced disease, chemotherapy is the key means to achieve the treatment goal [10, 11]. However, the inherent cytotoxicity of chemotherapy drugs kills cancer cells while inevitably damaging the rapidly proliferating normal tissues of the body, which can trigger various adverse events.

Among the many adverse reactions to chemotherapy, chemotherapy-induced nausea and vomiting (CINV) represents one of the most intractable clinical problems due to its high prevalence, significant distress, and serious impact on treatment compliance and overall well-being [12]. Chemotherapeutic regimens for BC, particularly highly emetogenic agents such as anthracycline-cyclophosphamide (AC/EC) and moderately emetogenic combinations such as carboplatin or taxanes, frequently induce CINV [13]. Notably, the acute-phase CINV rate for some drugs exceeds 90% [13]. The mechanism of CINV is complex, involving peripheral gastrointestinal tract injury (such as 5-HT release), central chemoreceptor trigger zone (CTZ) activation and cerebral cortex regulation (such as anticipatory response) [14].

CINV not only causes serious physical discomfort (such as dehydration, electrolyte disturbance and malnutrition) but also induces deep psychological distress (such as anxiety and depression), and even forces patients to consider interrupting treatment, which directly affects the implementation of adequate chemotherapy and the final efficacy [15]. Therefore, effective prevention and management of CINV is the key link to facilitate the successful administration of BC chemotherapy and maintain life quality among this population. However, the current understanding of the risk factors of CINV is still controversial. In this context, this systematic review and meta-analysis aims to evaluate the prevalence and risk factors of CINV in BC patients. This will help clinicians to identify high-risk patients early, thereby enabling the implementation of more effective preventive strategies to alleviate patient suffering and improve the treatment experience.

## Patients/material and methods

### Materials and methods

The approach was designed in line with the PRISMA-P guidelines for systematic review and meta-analysis protocols. The review will be carried out according to PRISMA guidelines [16]. Registration number: CRD420251123006. The PRISMA 2020 checklist is available in the Supplementary materials.

### Literature retrieval

Several databases, including CNKI, Wanfang, VIP databases, PubMed, Embase, Cochrane, Web of Science and others, were systematically queried to identify research articles reporting the prevalence and risk factors of CINV in BC patients. The retrieval time was up to July 1, 2025. Subject words plus free words were used to search: Breast Neoplasms, Nausea, Vomiting, and Risk Factors. Detailed search approaches are provided in the Supplementary Material.

### Inclusion and exclusion criteria

The literature selection criteria were established following the PICOS (Population, Intervention, Comparison, Outcomes and Study) framework [16, 17]. The included studies were required to focus on patients with pathologically confirmed BC undergoing chemotherapy, to investigate the prevalence and risk factors of CINV. We pre-specified that quantitative data synthesis would only be carried out when a risk factor was reported by at least three independent studies. The exposure factor is that the patient reports nausea and vomiting once or more during chemotherapy. Eligible studies must report the prevalence of nausea and vomiting and provide odds ratios (ORs) with 95% confidence intervals (CIs). Eligible study designs included observational studies, such as cohort and cross-sectional studies. Furthermore, the following were excluded: studies for which the full text was unavailable; studies that did not analyze relevant risk factors; conference abstracts; meta-analyses; protocols; letters; systematic reviews; republished articles; and animal experiments. These criteria were implemented to ensure the quality and relevance of the final included literature.

### Data extraction

The literature screening and data extraction process was independently completed by two researchers. Firstly, the researchers independently reviewed the titles and abstracts of all retrieved literature and initially screened out the studies that met the inclusion criteria. Subsequently, the full texts of these studies were obtained for detailed evaluation to determine the studies ultimately included in the analysis. The extracted data content includes: the name of the first author, publication year, country, research design, sample size, average age, and outcome indicators. After the two researchers independently complete the extraction, they will conduct cross-checks. In case of any disagreement, they will resolve it through consultation and discussion. If necessary, a third researcher will be consulted to make a decision.

### Quality evaluation

The methodological quality of cohort studies was assessed using the Newcastle-Ottawa Scale (NOS), which evaluates three domains: selection of study groups (up to 4 points), comparability of cohorts (up to 2 points), and ascertainment of either exposure or outcome (up to 3 points). The quality score ranges from 0 to 9, with a score of 7 or above representing high quality [18]. The Agency for Healthcare Research and Quality (AHRQ) tool was used to assess the quality of the cross-sectional studies [18]. This instrument consists of 11 items, each rated as ‘yes’, ‘no’, or ‘unclear’. Any discrepancies among evaluators were resolved through discussion or by consultation with a third reviewer.

### Statistical analysis

This study conducted a meta-analysis using Stata 15.1 to evaluate the associations between multiple factors (such as age, history of motion sickness, chemotherapy cycle, and anxiety status) and the risk of CINV. Specific data pooling strategies were formulated based on variable type. For continuous variables (e.g. age), we extracted the mean and standard deviation to calculate the standardized mean difference. If a continuous variable was reported as dichotomous, we extracted its OR and 95% CI. For inherent dichotomous variables, the OR and 95% CI were used directly for effect size pooling. Heterogeneity among studies was evaluated using the Q-test and the *I*^2^ statistic. The selection of a fixed-effect or random-effects model was predetermined based on heterogeneity thresholds outlined in the Cochrane Handbook (https://www.cochrane.org/authors/handbooks-and-manuals/handbook). A random-effects model was employed when substantial heterogeneity was detected (*I*² ≥ 50% or a Cochran’s Q test *P*-value < 0.10); otherwise, a fixed-effect model was applied. Sensitivity analysis or subgroup analysis was conducted for analyses with high heterogeneity to identify potential factors influencing the pooled effect size. Publication bias was assessed through visual inspection of funnel plots and Egger’s test (*p* < 0.05 was considered indicative of significant bias), with the trim-and-fill method employed for confirmation when necessary. The results are presented as pooled ORs with 95% CIs, and *p* < 0.05 was considered statistically significant.

## Results

### Overview of included studies

**Figure 1:**
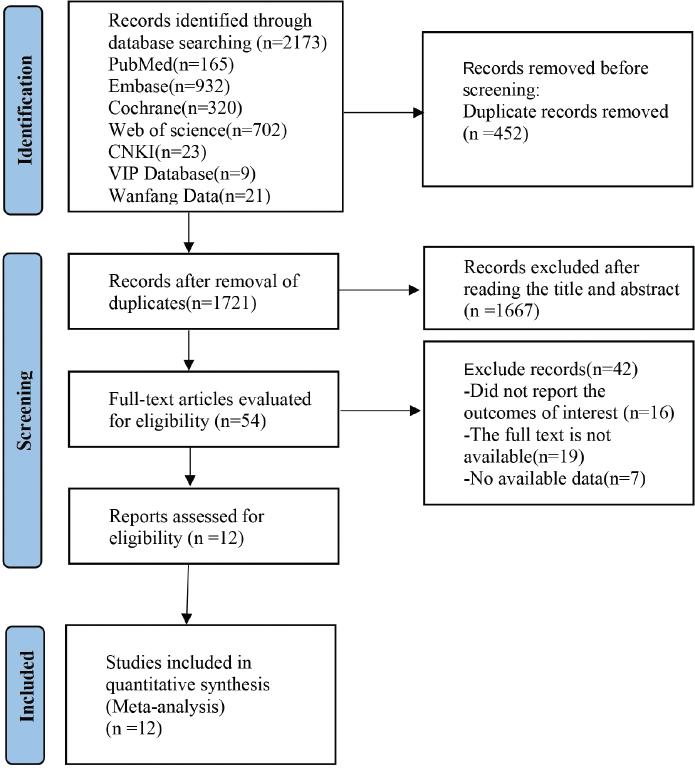
Flowchart of literature retrieval.

A total of 2,173 records were initially identified through the literature search. Following screening, 12 articles were included in the systematic review [19–30], and the specific search flow diagram is shown in Figure 1. These studies encompassed 4,200 BC patients undergoing chemotherapy, among whom 1,682 experienced post-chemotherapy nausea and vomiting. The basic characteristics of the included studies are summarized in Table 1. The potential risk factors available for analysis comprised age, history of motion sickness, Chemotherapy cycle ≥ 3, anxiety, and comorbidities. Finally, the following factors that can be used to analyze potential risk factors were identified and defined:

Age: Categorized as ≤ 45 years or > 45 years.

History of motion sickness: Determined by patient self-report or prior medical records.

Chemotherapy cycle: Defined as the receipt of ≥ 3 chemotherapy cycles.

Anxiety: Defined by a Self-Rating Anxiety Scale (SAS) score of ≥ 50 [31].

Comorbidities: The presence of any underlying disease, such as cardiovascular disease, diabetes, or chronic kidney disease.

**Table 1:** Basic information included in the study.

Study	Year	County	Study design	Sample size	No of NV	Mean age	Regression model
W. Yao	2017	China	Cohort study	80	69	45	NR
X.Y. Lu	2023	China	Cohort study	339	134	55	Multivariate logistic regression
H.L. Ying	2022	China	Cohort study	88	21	50.17 ± 5.32	Multivariate logistic regression
S.P. Quan	2018	China	Cohort study	100	65	44.5	Nr
X.C. Wei	2024	China	Cohort study	500	236	45	Multivariate logistic regression
Booth, C. M.	2007	Canada	Cohort study	143	51	51.4	Multivariate logistic regression
Roscoe, J. A.	2010	USA	Cohort study	1695	491	NR	Multivariate logistic regression
Nawa-Nishigaki, M.	2018	Japan	Cohort study	73	66	55.45	Multivariate logistic regression
Huang, X.	2021	China	Cohort study	400	213	NR	Multivariate logistic regression
Singh, K. P.	2022	USA	Cohort study	532	251	54.5	Multivariate logistic regression
Ng, B.	2023	Indonesia	Cross-sectional	137	59	52.1	Multivariate logistic regression
Jiang, T.	2025	China	Cohort study	113	26	51.66 ± 9.91	Multivariate logistic regression

NV: Nausea and Vomiting; NR: Not Reported.

### Quality assessment results

Regarding methodological quality, the 11 cohort studies all achieved NOS scores of ≥ 7, and the one cross-sectional study had an AHRQ score of 4, indicating an overall high quality of the included research. Supplementary Table S1 and Table S2 display the detailed quality assessment.

### The prevalence of nausea and vomiting in breast cancer patients after chemotherapy

**Figure 2:**
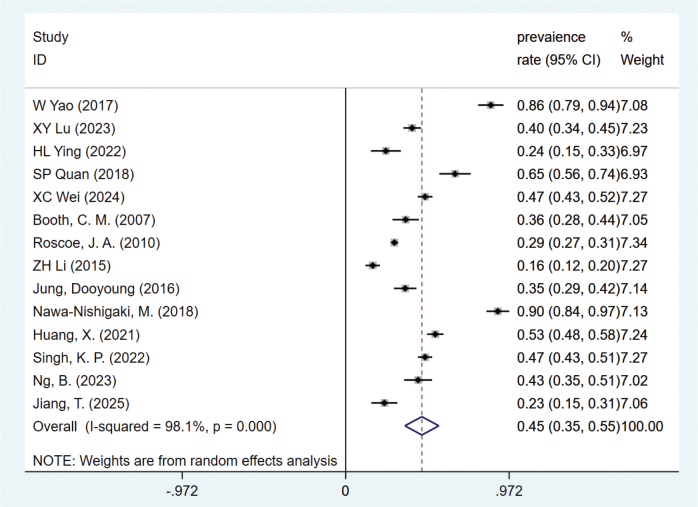
Meta-analysis forest plot of the prevalence of CINV in BC patients.

The prevalence of CINV in BC patients was extracted from 12 studies. Heterogeneity test (*I*^2^ = 98.1%, *P* = 0.000) was analyzed by random effects model, and the results (Figure 2) showed that the prevalence of nausea and vomiting in BC patients after chemotherapy was 49% (95% CI = 0.38–0.59). To evaluate the stability of this pooled estimate and to investigate the substantial heterogeneity observed, a sensitivity analysis was conducted. This analysis identified three studies whose effect sizes deviated significantly from the overall estimate (Supplementary Figure S1 in the Supplementary material). However, their removal only reduced the I² value from 98.1% to 91.3%, which remains substantially above the 50% threshold (Supplementary Figure S2). This indicates that these studies were not the primary sources of heterogeneity. Publication bias assessment (Supplementary Figure S3) showed that the funnel plot distribution was symmetric, and the Egger test showed *P* = 0.078, indicating that the possibility of publication bias of this index was small. In addition, we conducted subgroup analyses by country and study method. The CINV rates did not differ significantly across different methods. The overall prevalence of CINV was 48% (95% CI = 0.35–0.62) in China, 36% (95% CI = 0.28–0.44) in Canada, 38% (95% CI = 0.20–0.56) in the United States, and 90% (95% CI = 0.84–0.97) in Japan, as detailed in Supplementary Figures S4 and S5.

### Univariate meta-analysis

#### Age ≤ 45

Age was mentioned in five studies [24–27, 30], heterogeneity test (*I*^2^ = 83.6%, *P* = 0.000) was analyzed by random effect model, and the analysis results (Supplementary Figure S6) showed that age ≤ 45 was a risk factor for nausea and vomiting in BC patients after chemotherapy, with statistical significance (OR = 3.21, 95% CI = 1.44–7.17). Sensitivity analyses (Supplementary Figure S7) suggested that the sensitivity was modest and that the results were stable. Publication bias assessment showed that the funnel plot distribution was asymmetric (Supplementary Figure S8), and the Egger test showed that *P* = 0.049, indicating publication bias of this index. Further clipping and filling analysis showed that the combined effect size decreased and was no longer statistically significant after estimating and filling in two possibly missing studies (Supplementary Figure S9). The results may be overestimated by the existing publication bias.

#### History of motion sickness

Four studies mentioned the history of motion sickness [24–26, 30]. Heterogeneity test (I^2^ = 89.4%, P = 0.000) was analyzed by random effect model. The analysis results (Supplementary Figure S10 in the Supplementary Material) showed that the history of motion sickness was a risk factor for CINV in BC patients, with statistical significance (OR = 4.85, 95% CI = 1.65–14.30). Sensitivity analyses (Supplementary Figure S11) showed stable results when each study was removed individually. Publication bias assessment showed (Supplementary Figure S12) that the funnel plot distribution was symmetric, and the Egger test evaluation showed *P* = 0.898, indicating that the possibility of publication bias was small.

### Multivariate meta-analysis

#### Age ≤ 45

**Figure 3:**
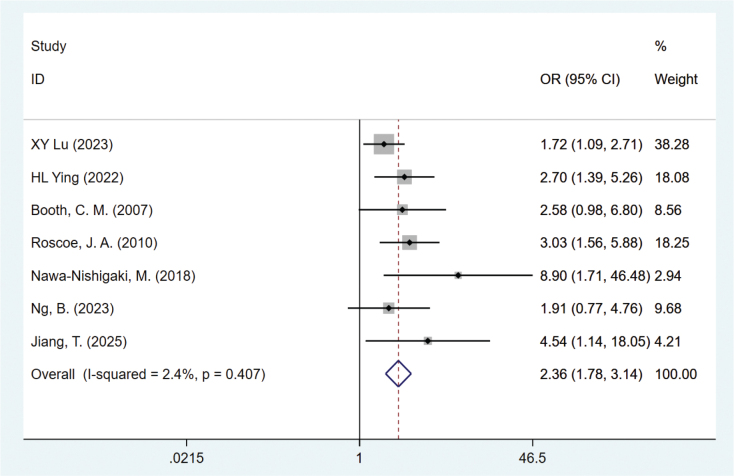
Forest plot of multivariate meta-analysis for age ≤ 45.

Seven studies mentioned age [19, 21–23, 25, 28, 30], heterogeneity test (*I*^2^ = 2.4%, *P* = 0.407) was analyzed by fixed-effect model, and the analysis results (Figure 3) showed that age ≤ 45 was a risk factor for nausea and vomiting in BC patients after chemotherapy, with statistical significance (OR = 2.36, 95% CI = 1.78–3.14). The funnel plot (Supplementary Figure S13) is roughly symmetrical visually, but Egger’s test *p* = 0.031, suggesting significant publication bias. Further trim-and-fill results indicated that the meta-analysis results were less likely to be affected by publication bias, as shown in Supplementary Figure S14.

#### History of motion sickness

**Figure 4:**
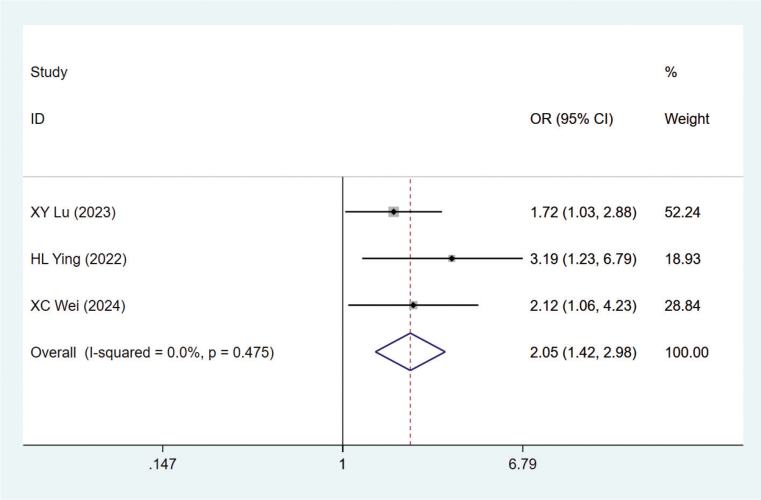
Forest plot of multivariate meta-analysis for motion sickness history.

Three studies mentioned the history of motion sickness [24, 25, 30]. Heterogeneity test (*I*^2^ = 0.0%, *P* = 0.475) was analyzed by fixed-effect model. The analysis results (Figure 4) showed that the history of motion sickness was a risk factor for CINV in BC patients, with statistical significance (OR = 2.05, 95% CI = 1.42–2.98). Moreover, the funnel plot (Figure S15) was approximately symmetrical and Egger’s test *p* = 0.138, suggesting no significant publication bias.

#### Chemotherapy cycle ≥ 3

**Figure 5:**
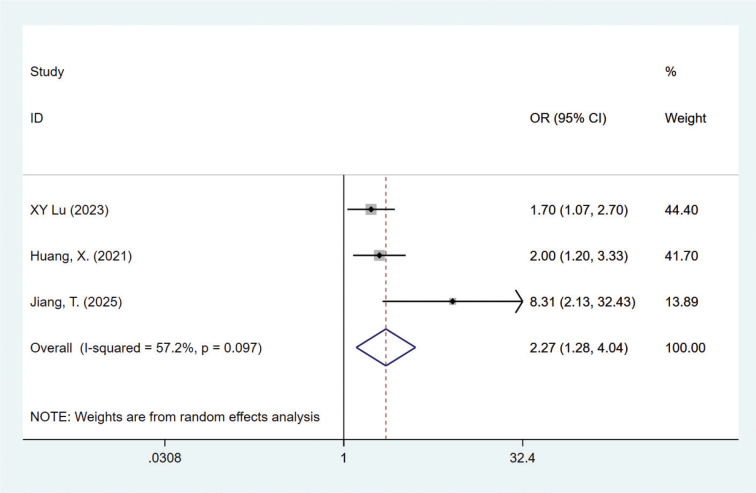
Forest plot of multivariate meta-analysis for chemotherapy cycles ≥ 3.

Three studies mentioned chemotherapy cycle [22, 25, 29], heterogeneity test (*I*^2^ = 57.2%, *P* = 0.097) was analyzed by random effect model, and the analysis results (Figure 5) showed that chemotherapy cycle ≥ 3 was a risk factor for CINV in BC patients, with statistical significance (OR = 2.27, 95% CI = 1.28–4.04). Sensitivity analysis (Figure S16) showed that the results were stable after removing each study one by one. Publication bias assessment showed that the funnel plot distribution was asymmetric (Figure S17), but the Egger test evaluation showed *P* = 0.063, indicating a small possibility of publication bias.

### Other meta-analysis results

No significant associations were found between post-chemotherapy nausea and vomiting in BC patients and either anxiety (OR = 2.74, 95% CI = 0.66–11.29, *P* = 0.952) or comorbidities (OR = 1.04, 95% CI = 0.72–1.49, *P* = 0.649). See Figures S18 and S19 of the supplementary materials.]

## Discussion and conclusion

This is the first meta-analysis to thoroughly examine the prevalence and risk factors of CINV in individuals with BC. The analysis revealed a pooled CINV prevalence of 49%, with significant heterogeneity observed across the included studies. Although sensitivity and subgroup analyses were conducted (limited to country and methodological levels), they failed to identify the primary sources of heterogeneity. This suggests that the variation likely stems from unmeasured or unreported variables, such as chemotherapy cycles, specific drug protocols, antiemetic regimens, and outcome assessment criteria. Therefore, the pooled prevalence derived from this study should be considered preliminary evidence. Future research should focus on prospective studies with unified key variables and standardized evaluation criteria to obtain more consistent and reliable evidence.

This study found that age was a risk factor for CINV in BC patients, and younger age was associated with a significantly increased risk of CINV. This finding is consistent with the results of a recently published systematic review of CINV risk prediction models, which clearly listed ‘young age’ as one of the top 10 most frequently reported predictors [32]. As for the reasons behind this phenomenon, we speculate that compared with young patients, elderly patients have rich life experience, strong psychological endurance, decreased body indicators, lower stimulation level, attenuated physical function, and slower conditioning reflex, and the overall response intensity of the body to the stimulation caused by chemotherapy drugs tends to be relaxed, thereby reducing the risk of CINV [33]. Therefore, special attention should be paid to younger patients, with more aggressive prevention strategies in the selection of an antiemetic regimen, and psychosocial support can be introduced early [34–36].

This study also found that the history of motion sickness was a predictor of CINV in BC patients [37, 38]. These results were similar to those in previous studies [37, 38]. A proposed mechanism is that motion sickness and CINV share common neural pathways. This shared pathophysiology centers on a hypersensitive vestibular system and a lowered excitation threshold in brainstem integration centers, particularly the area postrema and the nucleus tractus solitaries [39, 40]. In susceptible individuals, sensory mismatch readily activates this primed vestibular system, triggering the release of emetogenic neurotransmitters such as histamine and acetylcholine [39, 40]. During chemotherapy, cytotoxic drugs and their associated gastrointestinal reactions act as potent stimuli. These stimuli can amplify nausea and vomiting through shared or overlapping neural pathways, resulting in CINV that is more easily triggered and more severe [41]. In clinical practice, a history of motion sickness can be used as a simple and effective screening tool to identify people at high risk of CINV. For such patients, intensive intervention is recommended in addition to standard antiemetic regimens. Specific strategies include adding neuromodulators (such as olanzapine) or vestibular pathway antihistamines. This multipronged approach allows for more targeted prevention and management of CINV.

In this study, meta-analysis confirmed that undergoing ≥ 3 chemotherapy cycles was a significant risk factor for CINV. This finding appears to contradict some studies that identify it as a protective factor, but it actually reveals the dynamic evolution of CINV risk [29, 38]. For example, Huang et al. found that ≥ 3 cycles served as a protective factor (OR = 0.5) in a single-cycle observation. This may reflect physiological adaptation and ‘survivor bias’ (i.e. patients who tolerate early treatment proceed to later cycles) [29]. However, the conclusions of this study are more consistent with studies emphasizing longitudinal risk: Failure to effectively control CINV in the first cycle is the strongest predictor of management failure in subsequent cycles [42]. Therefore, the identification of ≥ 3 cycles as a risk factor just highlights that the treatment process itself is an important marker of cumulative risk and refractory CINV. The above-mentioned differences are unified in the dual implications of clinical practice. First of all, the most effective prevention must be carried out from the first cycle to break the vicious cycle of ‘control failure – increased risk’. Secondly, for patients who have entered multiple treatment cycles (≥ 3 cycles), if the CINV problem persists, they should be regarded as refractory patients, and the antiemetic regimen should be upgraded or adjusted in a timely manner. In conclusion, these two perspectives jointly point out that the prevention and treatment of CINV must be both forward-looking and individualized, and precise intervention should be adopted based on the risk characteristics of different treatment stages.

Meanwhile, anxiety and comorbidities may be associated with CINV, although the combined effect was not statistically significant in this study. This finding provides a valuable direction for improving the CINV risk prediction model. Therefore, we suggest that future models adopt a hierarchical integration strategy. Firstly, well-established factors (such as age and chemotherapy regimen) should form the core framework. Secondly, factors requiring further validation (such as anxiety and comorbidities) should be incorporated as subgroup correction variables. Finally, novel factors that have not been fully studied (including adherence to antiemetic medication, administration time windows, specific gene polymorphisms, and the quality of doctor–patient communication) should be actively explored. Clinicians should jointly consider these identified risk factors to facilitate the rapid identification of high-risk patients before chemotherapy. This enables early intervention and the formulation of personalized antiemetic prophylaxis plans, which helps to improve treatment outcomes and patient quality of life, as well as promote the rational allocation of antiemetic drugs and medical resources.

This study has several deficiencies that need to be taken into account when interpreting the results. (1) The number of included articles is limited, and most of the studies are from China, which may have selection bias. (2) The heterogeneity of some analysis results was significant, and the sensitivity analysis failed to fully explain it. (3) The assessment criteria for some risk factors (such as Comorbidities) are inconsistent in some studies, which may lead to measurement bias and heterogeneity. (4) Many of the included studies relied on patient-reported questionnaires to assess CINV, an approach that may result in inaccurate symptom reporting. (5) There is a lack of key clinical data, including but not limited to specifics on the chemotherapy regimen, number of treatment cycles, treatment intent (such as neoadjuvant, adjuvant, or palliative), and antiemetic protocols. (6) Risk factors for nausea and vomiting were not analyzed separately, and the two may have differences in pathogenesis and clinical manifestations, which are necessary to be distinguished in future research.

### Conclusion

This meta-analysis found that the prevalence of CINV in patients with BC was 49%. Significant risk factors identified included age ≤ 45 years, a history of motion sickness, and the administration of three or more chemotherapy cycles. In contrast, anxiety and comorbidities were not statistically significant. Identifying high-risk patients based on these factors before initiating chemotherapy is crucial, as it enables clinicians to develop more targeted and comprehensive antiemetic strategies. However, the range of risk factors included in the current analysis remains limited. Future studies should therefore incorporate a broader spectrum of potential variables to better investigate how their combined effects influence CINV risk.

## Supplementary Material



## Data Availability

The data of the article is in the charts and supplementary materials.
