# Supplementary material for: Prevalence and risk factors for nausea and vomiting in breast cancer patients undergoing chemotherapy
Source: Acta Oncol. 2026 Apr 1;65:44933. doi: 10.2340/1651-226X.2026.44933 (PMC13051770; doi:10.2340/1651-226X.2026.44933)
Supplement: Supplementary file 1 [file AO-65-44933-s1.pdf]

PRISMA 2020 Checklist

	Item #	Checklist item	Location where item is reported
**TITLE**			
Title	1	Identify the report as a systematic review.	Page 1
**ABSTRACT**			
Abstract	2	See the PRISMA 2020 for Abstracts checklist.	Page 1
**INTRODUCTION**			
Rationale	3	Describe the rationale for the review in the context of existing knowledge.	Page 2-4
Objectives	4	Provide an explicit statement of the objective(s) or question(s) the review addresses.	Page 2-4
**METHODS**			
Eligibility criteria	5	Specify the inclusion and exclusion criteria for the review and how studies were grouped for the syntheses.	Page 5
Information sources	6	Specify all databases, registers, websites, organisations, reference lists and other sources searched or consulted to identify studies. Specify the date when each source was last searched or consulted.	Page 4
Search strategy	7	Present the full search strategies for all databases, registers and websites, including any filters and limits used.	Page 4
Selection process	8	Specify the methods used to decide whether a study met the inclusion criteria of the review, including how many reviewers screened each record and each report retrieved, whether they worked independently, and if applicable, details of automation tools used in the process.	Page 4
Data collection process	9	Specify the methods used to collect data from reports, including how many reviewers collected data from each report, whether they worked independently, any processes for obtaining or confirming data from study investigators, and if applicable, details of automation tools used in the process.	Page 4
Data items	10a	List and define all outcomes for which data were sought. Specify whether all results that were compatible with each outcome domain in each study	Page 5

	Item #	Checklist item	Location where item is reported
		were sought (e.g. for all measures, time points, analyses), and if not, the methods used to decide which results to collect.	
	10b	List and define all other variables for which data were sought (e.g. participant and intervention characteristics, funding sources). Describe any assumptions made about any missing or unclear information.	Page 5
Study risk of bias assessment	11	Specify the methods used to assess risk of bias in the included studies, including details of the tool(s) used, how many reviewers assessed each study and whether they worked independently, and if applicable, details of automation tools used in the process.	Page 6
Effect measures	12	Specify for each outcome the effect measure(s) (e.g. risk ratio, mean difference) used in the synthesis or presentation of results.	Page 7
Synthesis methods	13a	Describe the processes used to decide which studies were eligible for each synthesis (e.g. tabulating the study intervention characteristics and comparing against the planned groups for each synthesis (item #5)).	Page 7
	13b	Describe any methods required to prepare the data for presentation or synthesis, such as handling of missing summary statistics, or data conversions.	Page 7
	13c	Describe any methods used to tabulate or visually display results of individual studies and syntheses.	Page 7
	13d	Describe any methods used to synthesize results and provide a rationale for the choice(s). If meta-analysis was performed, describe the model(s), method(s) to identify the presence and extent of statistical heterogeneity, and software package(s) used.	Page 7
	13e	Describe any methods used to explore possible causes of heterogeneity among study results (e.g. subgroup analysis, meta-regression).	Page 7
	13f	Describe any sensitivity analyses conducted to assess robustness of the synthesized results.	Page 7
Reporting bias assessment	14	Describe any methods used to assess risk of bias due to missing results in a synthesis (arising from reporting biases).	Page 6
Certainty assessment	15	Describe any methods used to assess certainty (or confidence) in the body of evidence for an outcome.	Page 7
**RESULTS**			

	Item #	Checklist item	Location where item is reported
Study selection	16a	Describe the results of the search and selection process, from the number of records identified in the search to the number of studies included in the review, ideally using a flow diagram.	Page 8
	16b	Cite studies that might appear to meet the inclusion criteria, but which were excluded, and explain why they were excluded.	Page 8
Study characteristics	17	Cite each included study and present its characteristics.	Page 8
Risk of bias in studies	18	Present assessments of risk of bias for each included study.	Page 8
Results of individual studies	19	For all outcomes, present, for each study: (a) summary statistics for each group (where appropriate) and (b) an effect estimate and its precision (e.g. confidence/credible interval), ideally using structured tables or plots.	Page 8
Results of syntheses	20a	For each synthesis, briefly summarise the characteristics and risk of bias among contributing studies.	Page9-10
	20b	Present results of all statistical syntheses conducted. If meta-analysis was done, present for each the summary estimate and its precision (e.g. confidence/credible interval) and measures of statistical heterogeneity. If comparing groups, describe the direction of the effect.	Page9-10
	20c	Present results of all investigations of possible causes of heterogeneity among study results.	Page9-10
	20d	Present results of all sensitivity analyses conducted to assess the robustness of the synthesized results.	Page9-10
Reporting biases	21	Present assessments of risk of bias due to missing results (arising from reporting biases) for each synthesis assessed.	Page8
Certainty of evidence	22	Present assessments of certainty (or confidence) in the body of evidence for each outcome assessed.	Page10
**DISCUSSION**			
Discussion	23a	Provide a general interpretation of the results in the context of other evidence.	Page 10-13

	Item #	Checklist item	Location where item is reported
	23b	Discuss any limitations of the evidence included in the review.	Page 10-13
	23c	Discuss any limitations of the review processes used.	Page 10-13
	23d	Discuss implications of the results for practice, policy, and future research.	Page 10-13
**OTHER INFORMATION**			
Registration and protocol	24a	Provide registration information for the review, including register name and registration number, or state that the review was not registered.	Page 4
	24b	Indicate where the review protocol can be accessed, or state that a protocol was not prepared.	Page 4
	24c	Describe and explain any amendments to information provided at registration or in the protocol.	Page 4
Support	25	Describe sources of financial or non-financial support for the review, and the role of the funders or sponsors in the review.	Page 13
Competing interests	26	Declare any competing interests of review authors.	Page 13
Availability of data, code and other materials	27	Report which of the following are publicly available and where they can be found: template data collection forms; data extracted from included studies; data used for all analyses; analytic code; any other materials used in the review.	Page 13

Search strategy

(((Breast Neoplasms[MeSH Terms]) OR ((((((((((((((((((((((((((((((((((((((Breast Neoplasms[Title/Abstract]) OR (Breast Neoplasm[Title/Abstract])) OR (Neoplasm, Breast[Title/Abstract])) OR (Neoplasms, Breast[Title/Abstract])) OR (Breast Tumors[Title/Abstract])) OR (Breast Tumor[Title/Abstract])) OR (Tumor, Breast[Title/Abstract])) OR (Tumors, Breast[Title/Abstract])) OR (Breast Cancer[Title/Abstract])) OR (Cancer, Breast[Title/Abstract])) OR (Cancer of Breast[Title/Abstract])) OR (Cancer of the Breast[Title/Abstract])) OR (Malignant Neoplasm of Breast[Title/Abstract])) OR (Breast Malignant Neoplasm[Title/Abstract])) OR (Breast Malignant Neoplasms[Title/Abstract])) OR (Malignant Tumor of Breast[Title/Abstract])) OR (Breast Malignant Tumor[Title/Abstract])) OR (Breast Malignant Tumors[Title/Abstract])) OR (Mammary Cancer[Title/Abstract])) OR (Cancer, Mammary[Title/Abstract])) OR (Cancers, Mammary[Title/Abstract])) OR (Mammary Cancers[Title/Abstract])) OR (Mammary Neoplasms, Human[Title/Abstract])) OR (Human Mammary Neoplasm[Title/Abstract])) OR (Human Mammary Neoplasms[Title/Abstract])) OR (Neoplasm, Human Mammary[Title/Abstract])) OR (Neoplasms, Human Mammary[Title/Abstract])) OR (Mammary Neoplasm, Human[Title/Abstract])) OR (Breast Carcinoma[Title/Abstract])) OR (Breast Carcinomas[Title/Abstract])) OR (Carcinoma, Breast[Title/Abstract])) OR (Carcinomas, Breast[Title/Abstract])) OR (Mammary Carcinoma, Human[Title/Abstract])) OR (Carcinoma, Human Mammary[Title/Abstract])) OR (Carcinomas, Human Mammary[Title/Abstract])) OR (Human Mammary Carcinomas[Title/Abstract])) OR (Mammary Carcinomas, Human[Title/Abstract])) OR (Human Mammary Carcinoma[Title/Abstract]))) AND (((Nausea[MeSH Terms]) OR (Vomiting[MeSH Terms])) OR (((Nausea[Title/Abstract]) OR (Vomiting[Title/Abstract])) OR (Emesis[Title/Abstract])))) AND ((Risk Factors[MeSH Terms]) OR (((((((((((((((((((Risk Factors[Title/Abstract]) OR (Factor, Risk[Title/Abstract])) OR (Risk Factor[Title/Abstract])) OR (Population at Risk[Title/Abstract])) OR (Populations at Risk[Title/Abstract])) OR (Risk Scores[Title/Abstract])) OR (Risk Score[Title/Abstract])) OR (Score, Risk[Title/Abstract])) OR (Risk Factor Scores[Title/Abstract])) OR (Risk Factor Score[Title/Abstract])) OR (Score, Risk Factor[Title/Abstract])) OR (Health Correlates[Title/Abstract])) OR (Correlates, Health[Title/Abstract])) OR (Social Risk Factors[Title/Abstract])) OR (Factor, Social Risk[Title/Abstract])) OR (Factors, Social Risk[Title/Abstract])) OR (Risk Factor, Social[Title/Abstract])) OR (Risk Factors, Social[Title/Abstract])) OR (Social Risk Factor[Title/Abstract])))

Table S1 Quality evaluation of NOS[1] in relevant cohort studies

Study	Representativeness of the exposed group	Selection of non-exposed groups	Determination of exposure factors	Identification of outcome indicators not yet to be observed at study entry	Comparability of exposed and unexposed groups considered in design and statistical analysis	design and statistical analysis	Adequacy of the study's evaluation of the outcome	Adequacy of follow-up in exposed and unexposed groups	Total scores
W Yao-2017	*	*	*	*	/	*	*	*	7
XY Lu-2023	*	*	*	*	*	*	*	*	8
HL Ying-2022	*	*	*	*	*	*	*	*	8
SP Quan-2018	*	*	*	*	/	*	*	*	7
XC Wei-2024	*	*	*	*	**	*	*	*	9
Booth, C. M-2007	*	*	*	*	**	*	*	*	9
Roscoe, J. A-2010	*	*	*	*	*	*	*	*	8
Nawa-Nishigaki, M-2018	*	*	*	*	**	*	*	*	9
Huang, X-2021	*	*	*	*	**	*	*	*	9
Singh, K. P-2023	*	*	*	*	*	*	*	*	8
Jiang, T-2025	*	*	*	*	*	*	*	*	8

[1] NOS: Newcastle-Ottawa Scale Quality Assessment

Table S2 Quality evaluation of AHRQ[2] in relevant cross-sectional studies

Study	Whether the source of the information is clear	Whether exposed and non-exposed groups are listed	Whether a time was given to identify patients	If not, population derived, whether the subjects were consecutive	Whether the subjective factors of the evaluator cover up other aspects of the research object	Any assessment performed to ensure quality is described	The rationale for excluding any patients from the analysis was explained	Describe measures to evaluate and/or control for confounding factors	explain how missing data were handled in the analysis	Response rates and the completeness of data collection are summarized	If there is follow-up, identify the percentage of patients with expected incomplete data or follow-up results
Ng, B-2023	Yes	No	Yes	Yes	Yes	unclear	unclear	unclear	unclear	unclear	unclear

[2] AHRQ: Agency for Healthcare Research and Quality;BMI:Body Mass Index

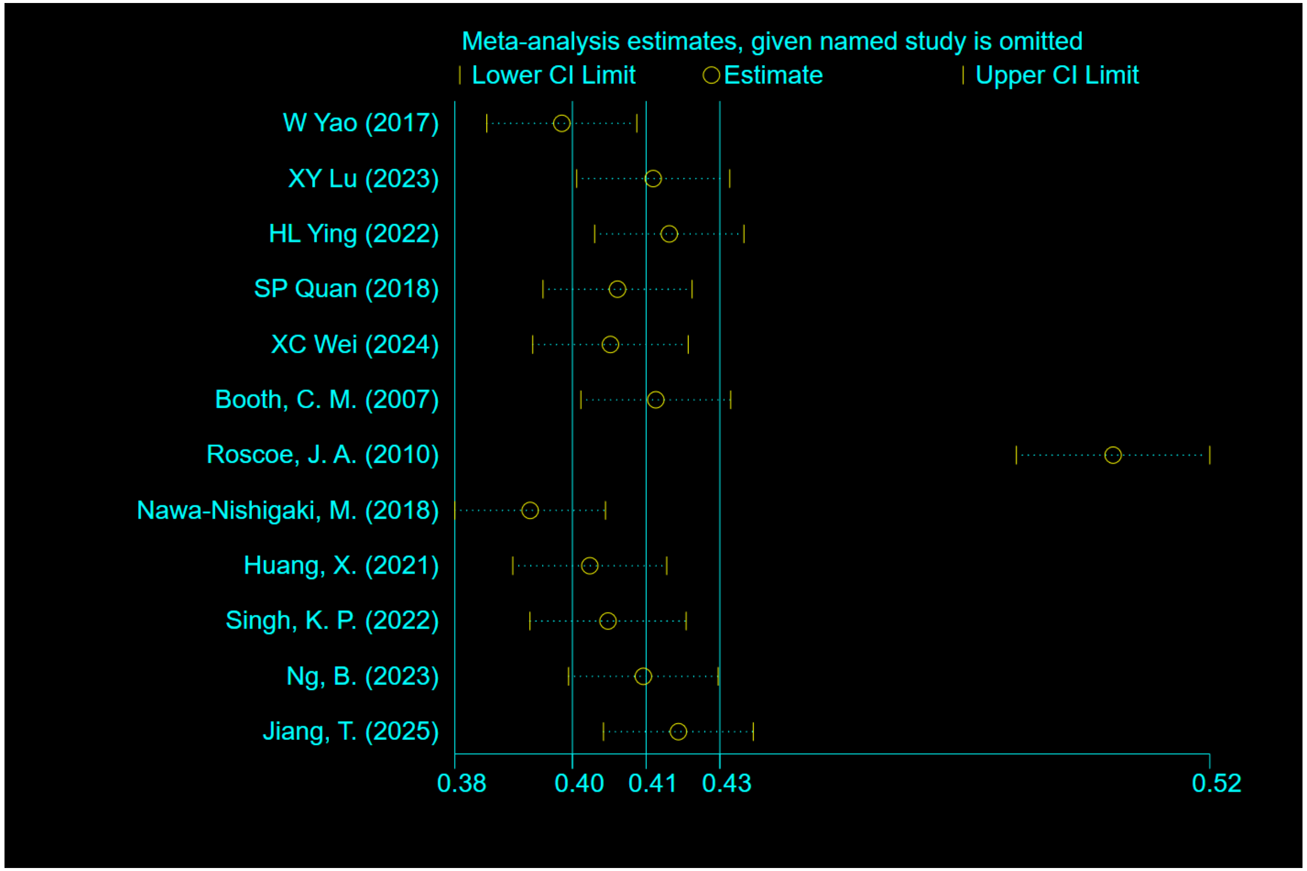

Figure S1 Sensitivity analysis of the prevalence of CINV in BC patients

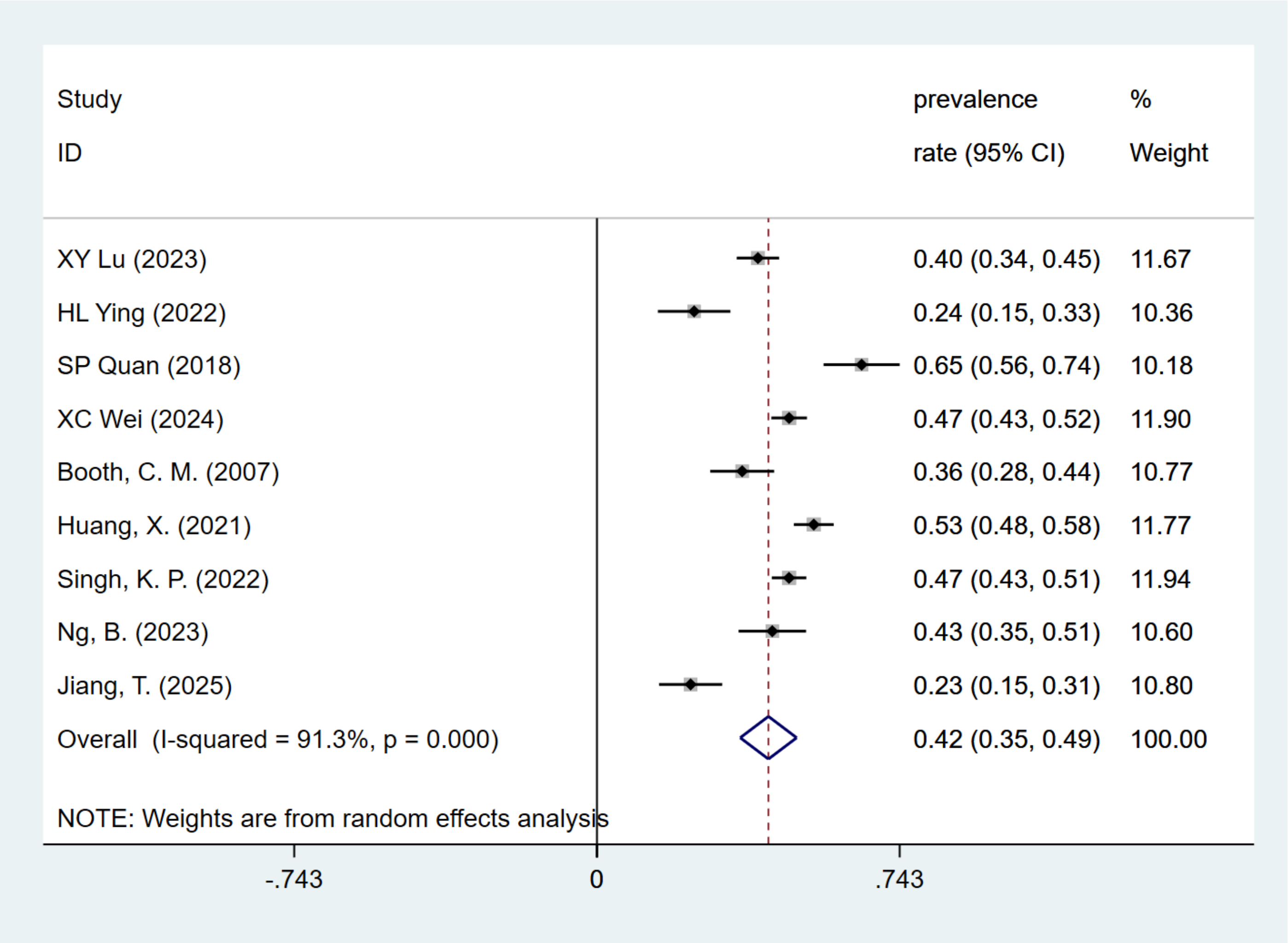

Figure S2 Forest plot after excluding three influential studies

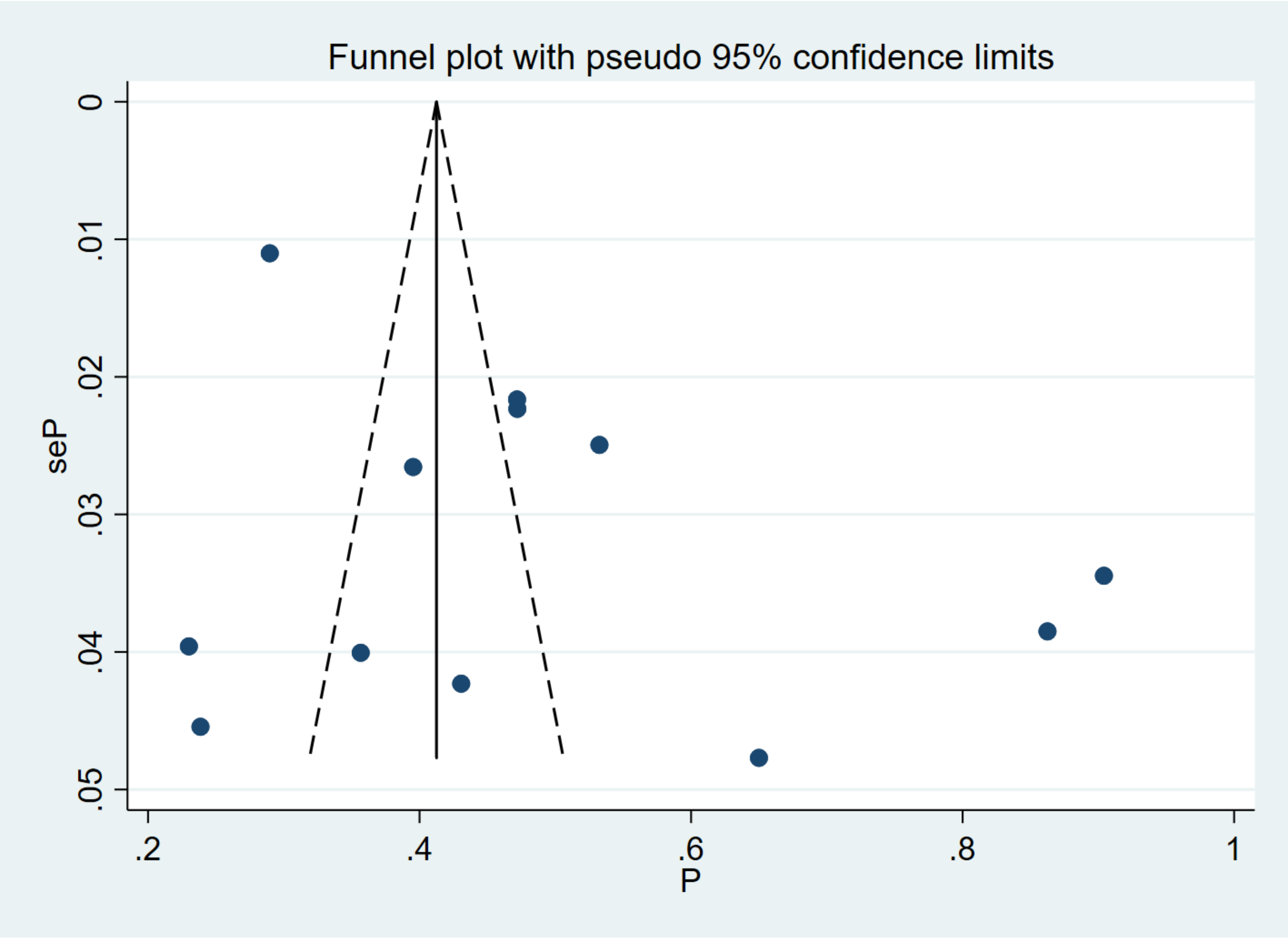

Figure S3 Meta-analysis funnel plot of CINV prevalence in BC patients

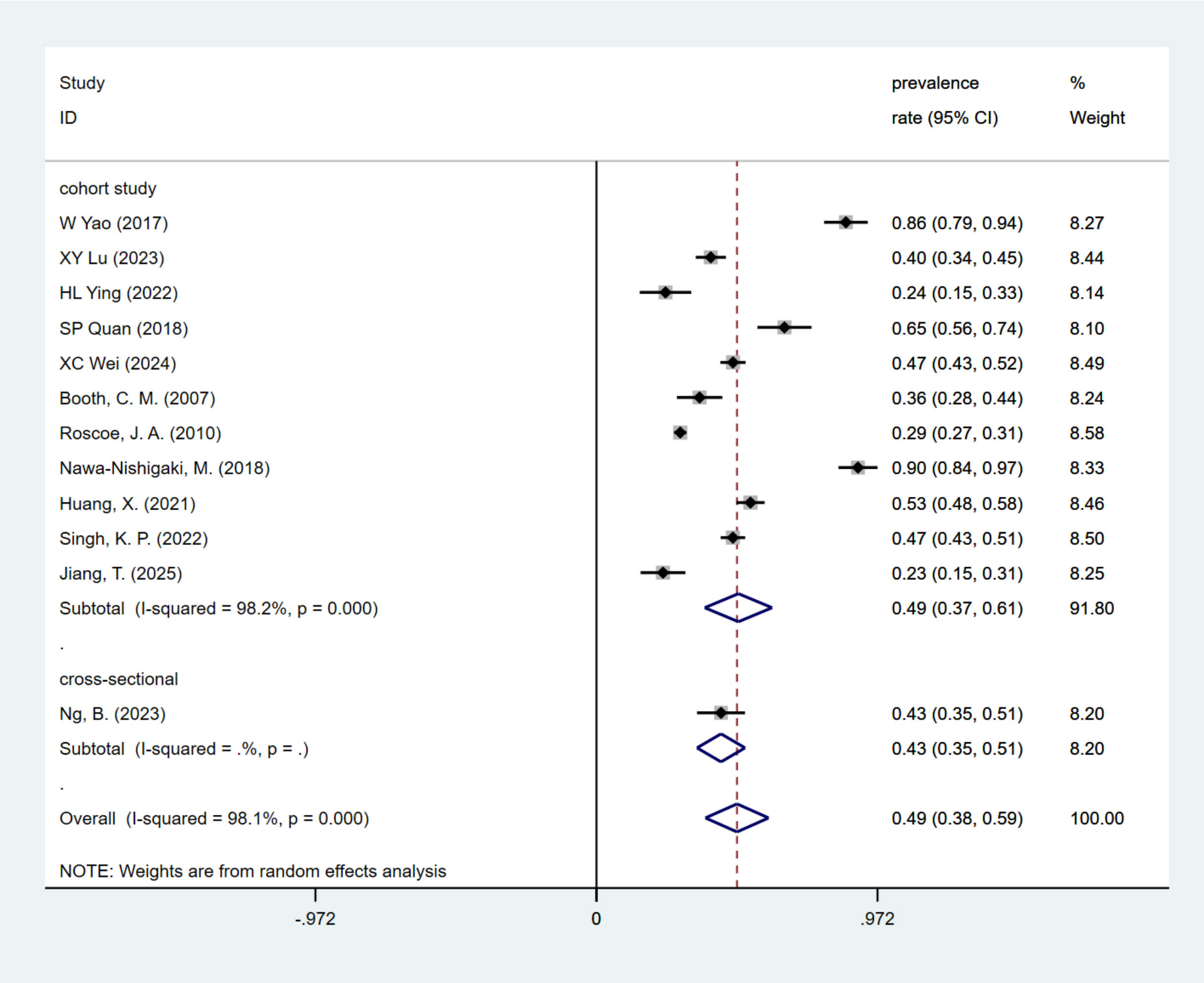

Figure S4 Subgroup analysis by study method for the prevalence of CINV in BC patients

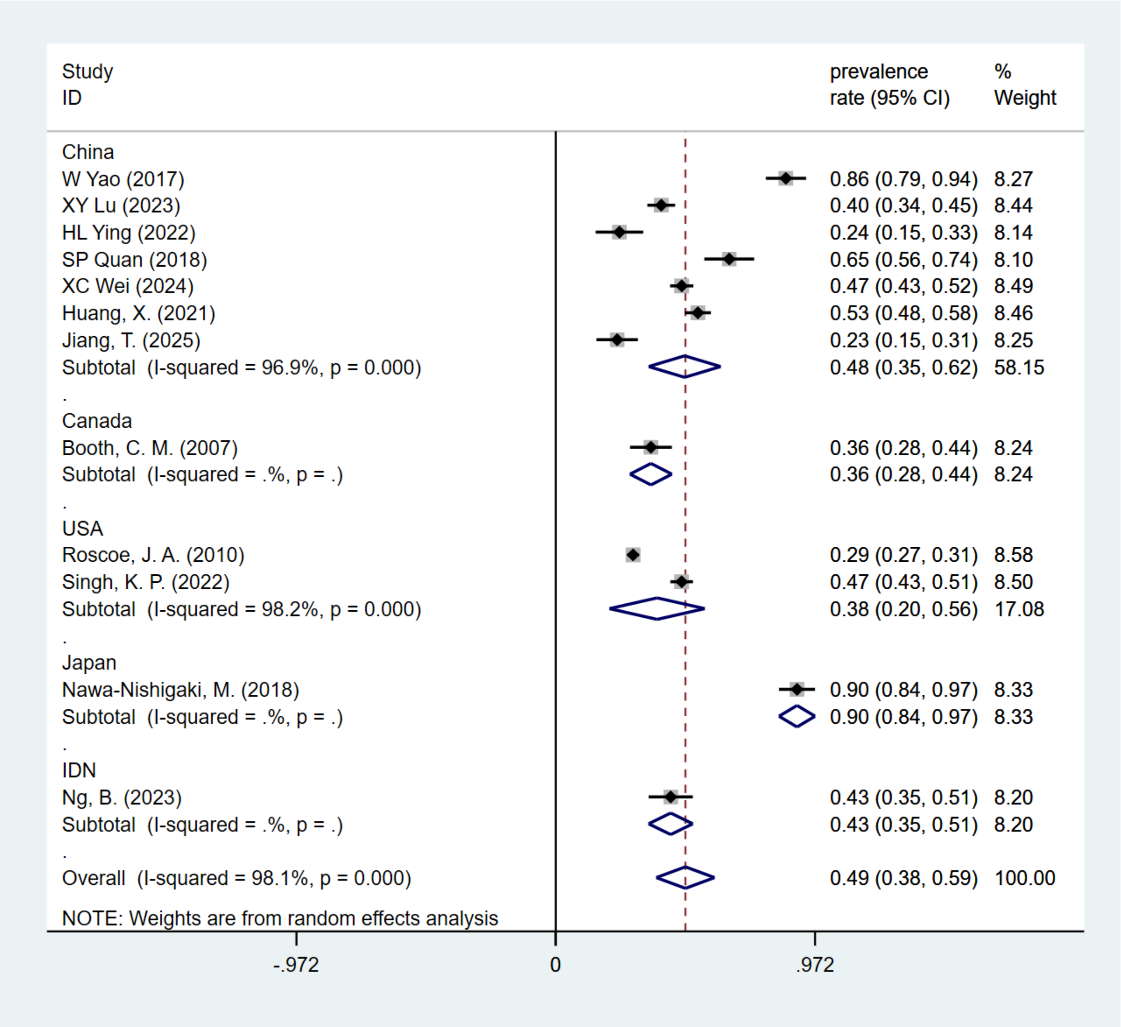

Figure S5 Subgroup analysis by country for the prevalence of CINV in BC patients

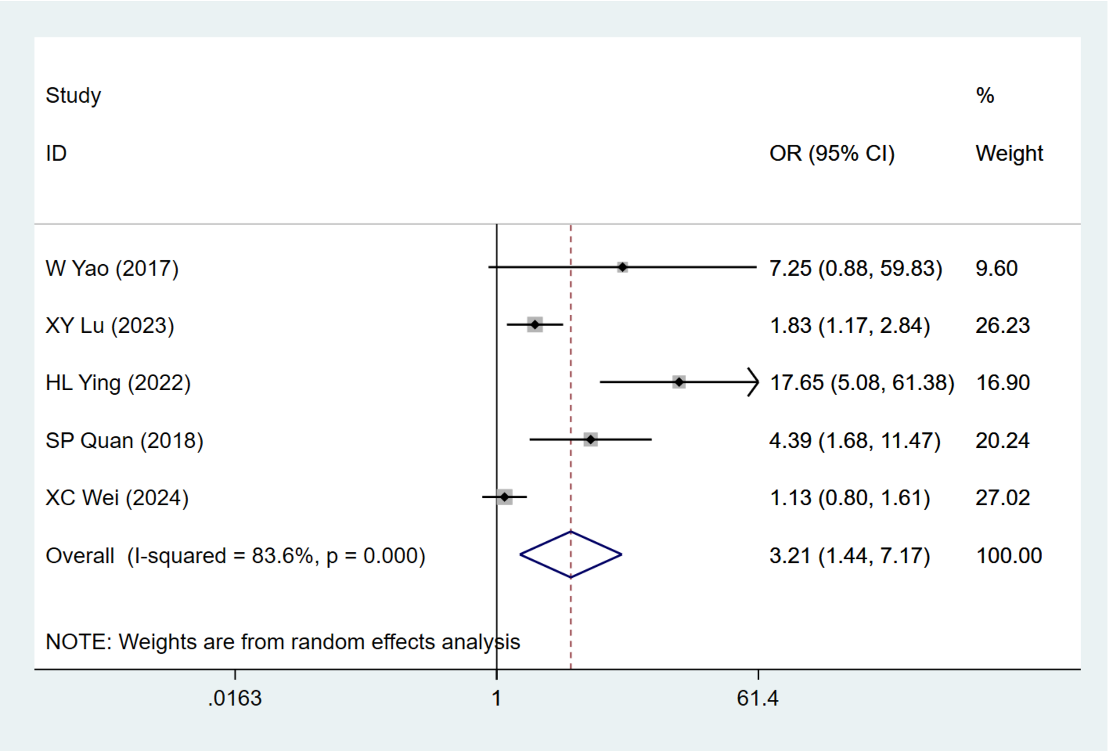

Figure S6 Forest plot of univariate meta-analysis for age ≤45

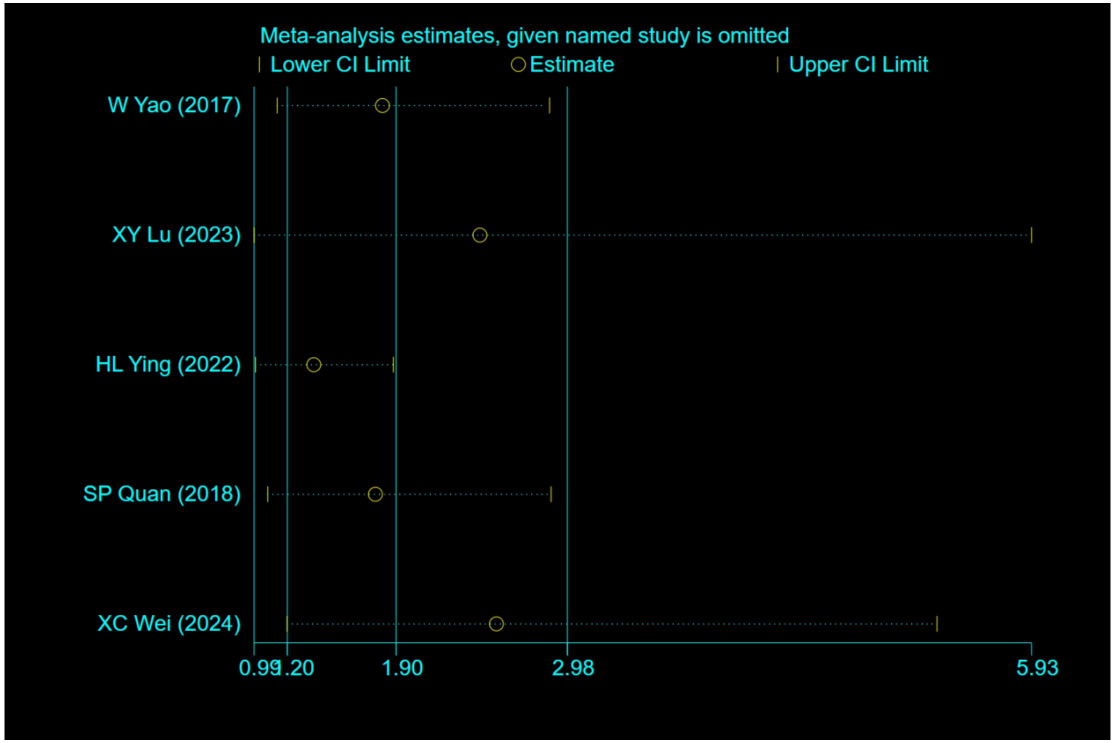

Figure S7 Sensitivity analysis of Age ≤45 (univariate meta-analysis)

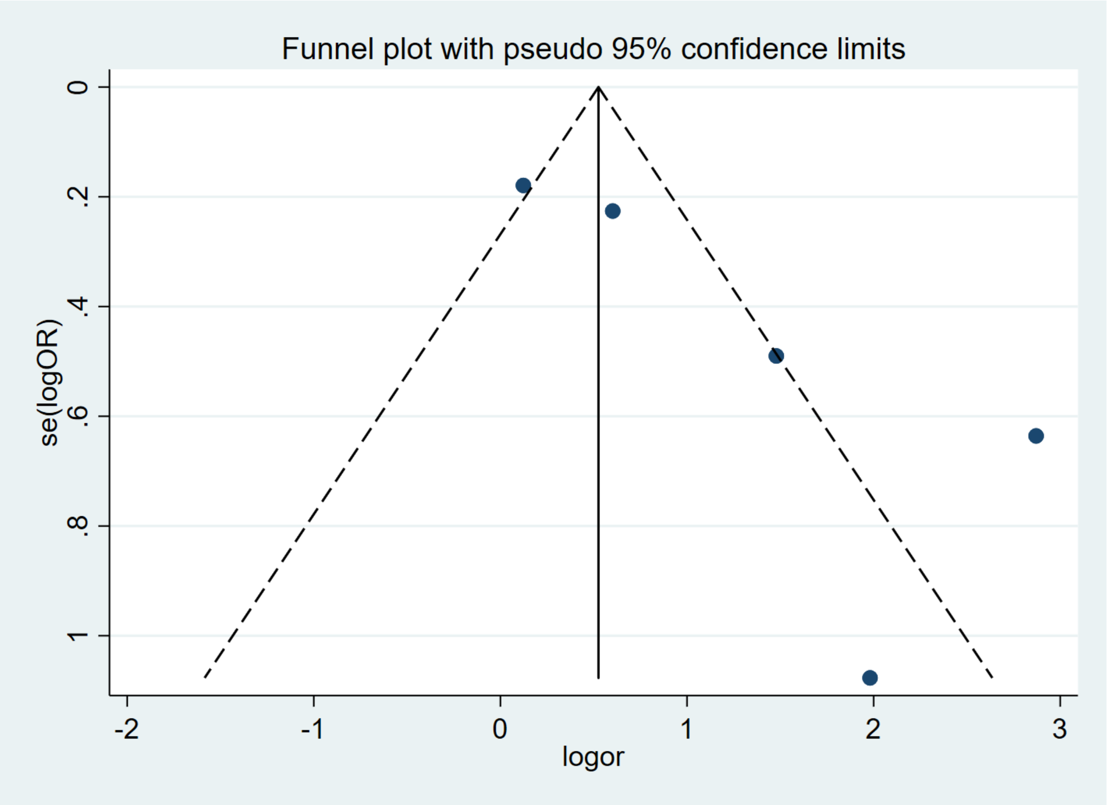

Figure S8 Funnel plot of Age ≤45 year (univariate meta-analysis)

```
. metatrim logor _selogES, reffect funnel

Note: default data input format (theta, se_theta) assumed.

Meta-analysis

       |  Pooled      95% CI         Asymptotic      No. of
Method |     Est   Lower   Upper  z_value  p_value   studies
-------+----------------------------------------------------
Fixed  |   0.526   0.269   0.783   4.010    0.000       5
Random |   1.166   0.363   1.969   2.845    0.004

Test for heterogeneity: Q= 24.369 on 4 degrees of freedom (p= 0.000)
Moment-based estimate of between studies variance =  0.589

Trimming estimator: Linear
Meta-analysis type: Random-effects model

iteration |  estimate    Tn    # to trim     diff
----------+--------------------------------------
    1     |    1.166      9          1         15
    2     |    0.703     12          2          6
    3     |    0.596     13          2          2
    4     |    0.596     13          2          0

Filled
Meta-analysis

       |  Pooled      95% CI         Asymptotic      No. of
Method |     Est   Lower   Upper  z_value  p_value   studies
-------+----------------------------------------------------
Fixed  |   0.419   0.169   0.668   3.283    0.001       7
Random |   0.644  -0.155   1.442   1.581    0.114

Test for heterogeneity: Q= 37.180 on 6 degrees of freedom (p= 0.000)
Moment-based estimate of between studies variance =  0.798
```

Figure S9 Trim-and-fill analysis of age ≤45 years (univariate meta-analysis)

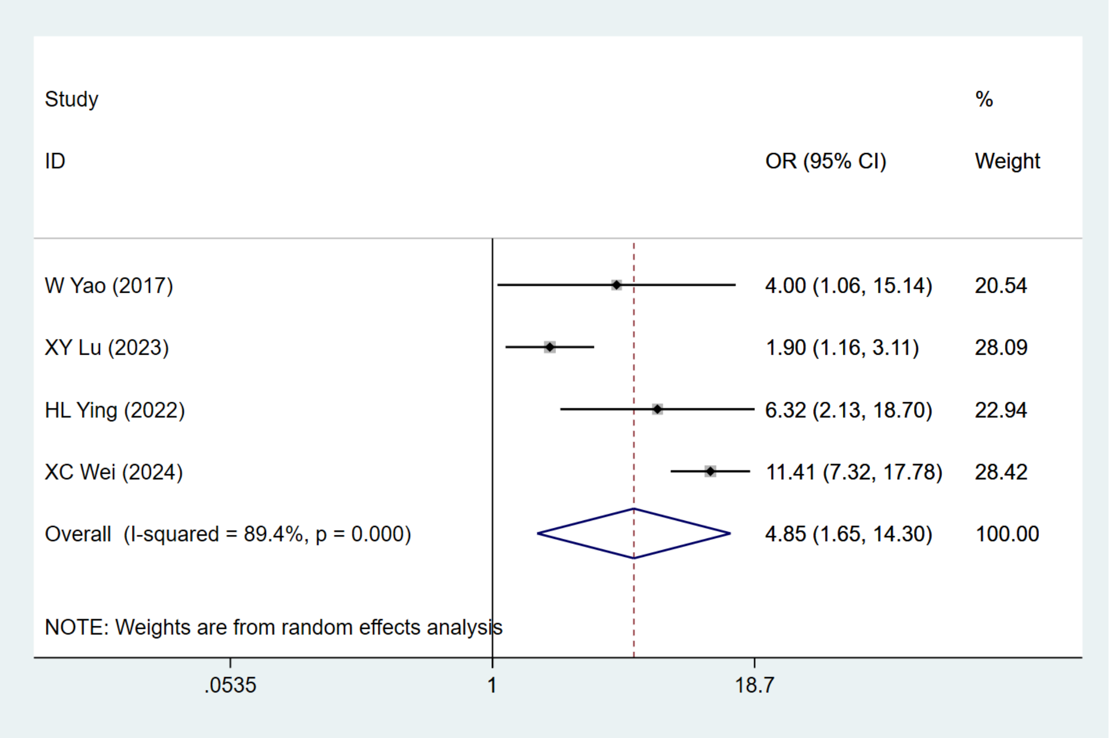

Figure S10 Forest map of motion sickness history (univariate meta-analysis)

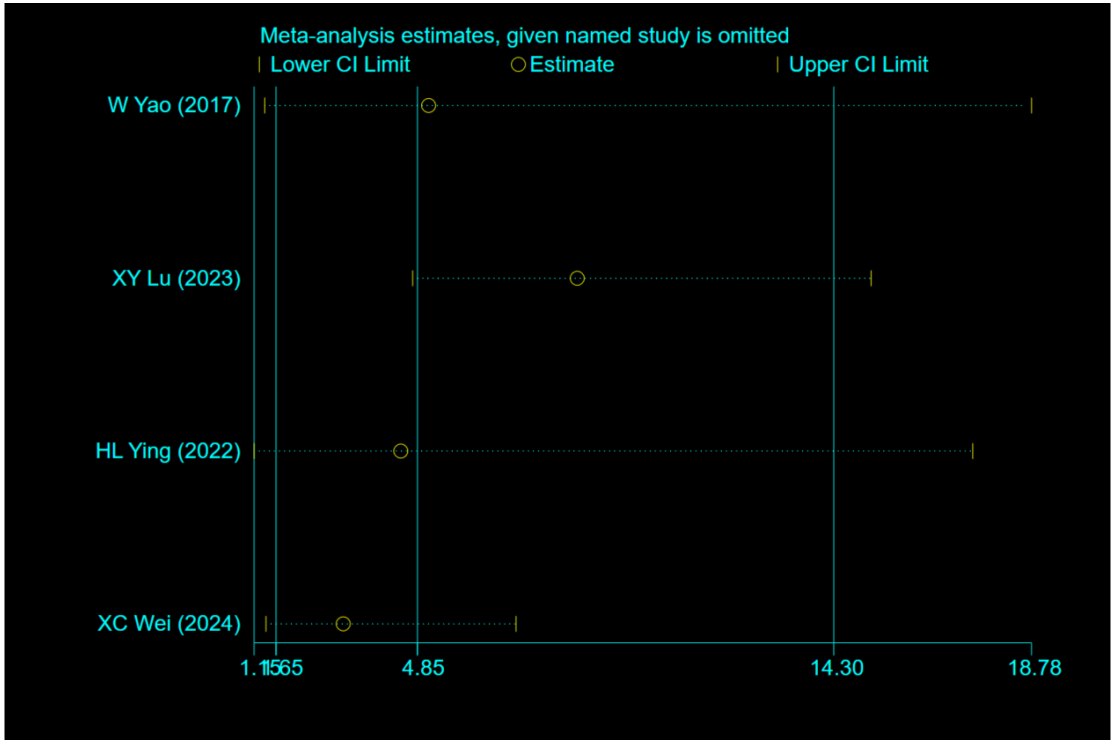

Figure 11 Sensitivity analysis of Motion sickness history (univariate meta-analysis)

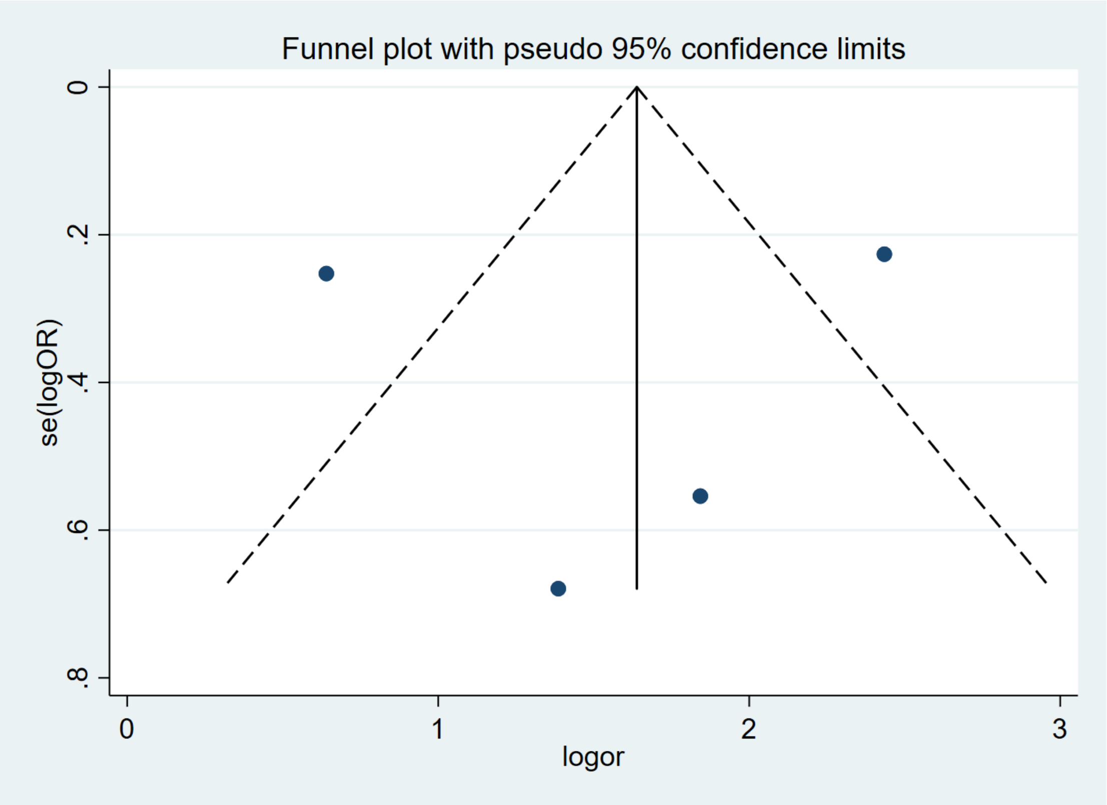

Figure S12 Funnel plot of History of motion sickness (univariate meta-analysis)

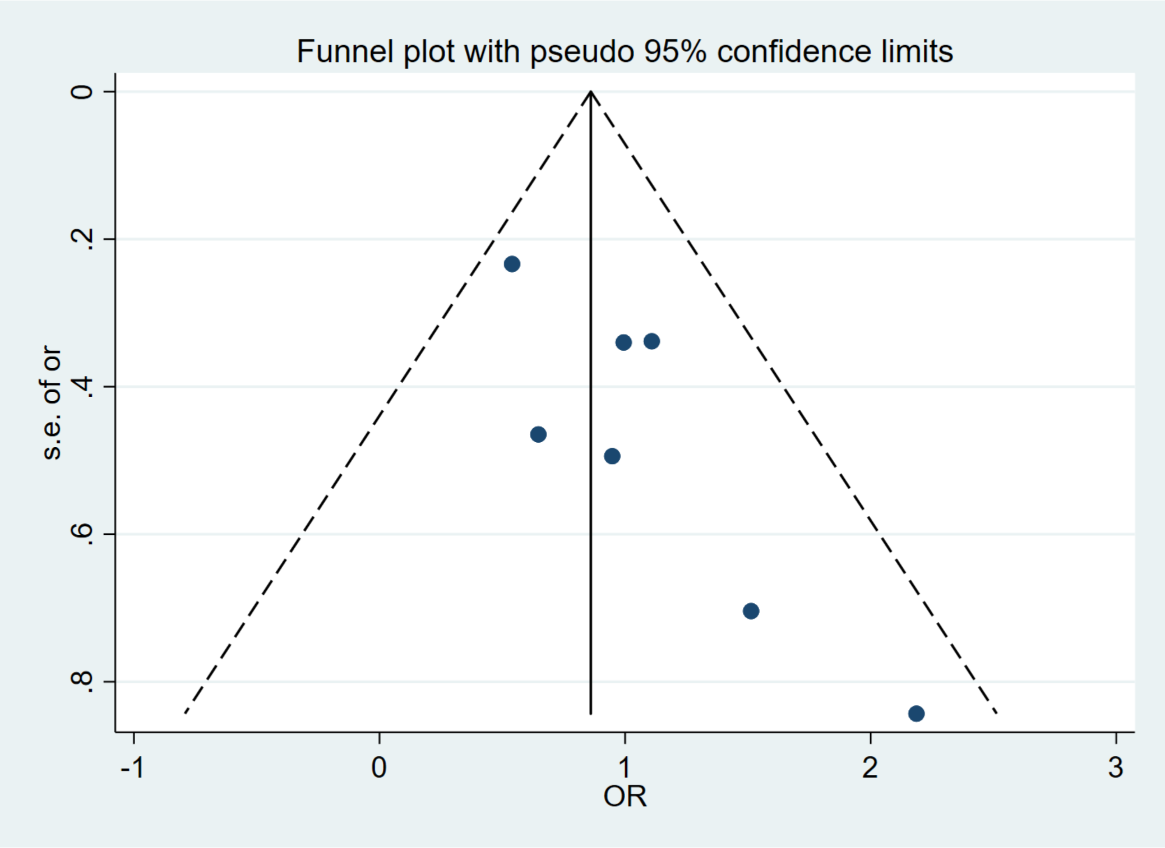

Figure S13 Funnel plot of Age ≤45 (multivariate meta-analysis)

```
. metatrim or lci uci, ci funnel

Note: option 'ci' specified.

Meta-analysis

       |  Pooled      95% CI         Asymptotic      No. of
Method |     Est    Lower   Upper  z_value  p_value   studies
-------+----------------------------------------------------
Fixed  |   0.861    0.577   1.144    5.952    0.000       7
Random |   0.867    0.577   1.156    5.867    0.000

Test for heterogeneity: Q=  6.147 on 6 degrees of freedom (p= 0.407)
Moment-based estimate of between studies variance =  0.004

Trimming estimator: Linear
Meta-analysis type: Fixed-effects model

iteration |  estimate    Tn    # to trim     diff
----------+--------------------------------------
    1     |    0.861     20         2          28
    2     |    0.789     23         3           6
    3     |    0.711     25         3           4
    4     |    0.711     25         3           0

Filled
Meta-analysis

       |  Pooled      95% CI         Asymptotic      No. of
Method |     Est    Lower   Upper  z_value  p_value   studies
-------+----------------------------------------------------
Fixed  |   0.711    0.458   0.964    5.505    0.000      10
Random |   0.730    0.401   1.060    4.343    0.000

Test for heterogeneity: Q= 12.964 on 9 degrees of freedom (p= 0.164)
Moment-based estimate of between studies variance =  0.080
```

Figure S14 Trim-and-fill analysis of age ≤45 years (multivariate meta-analysis)

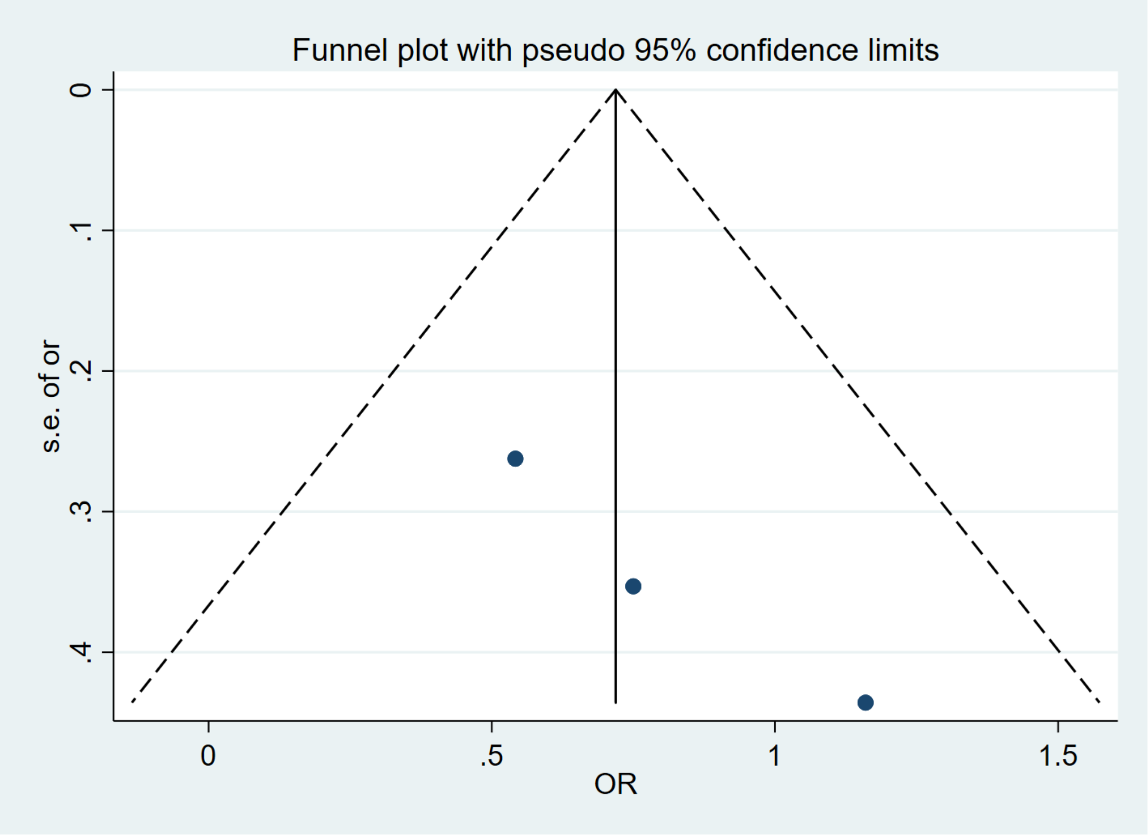

Figure S15 Funnel plot of History of motion sickness (multivariate meta-analysis)

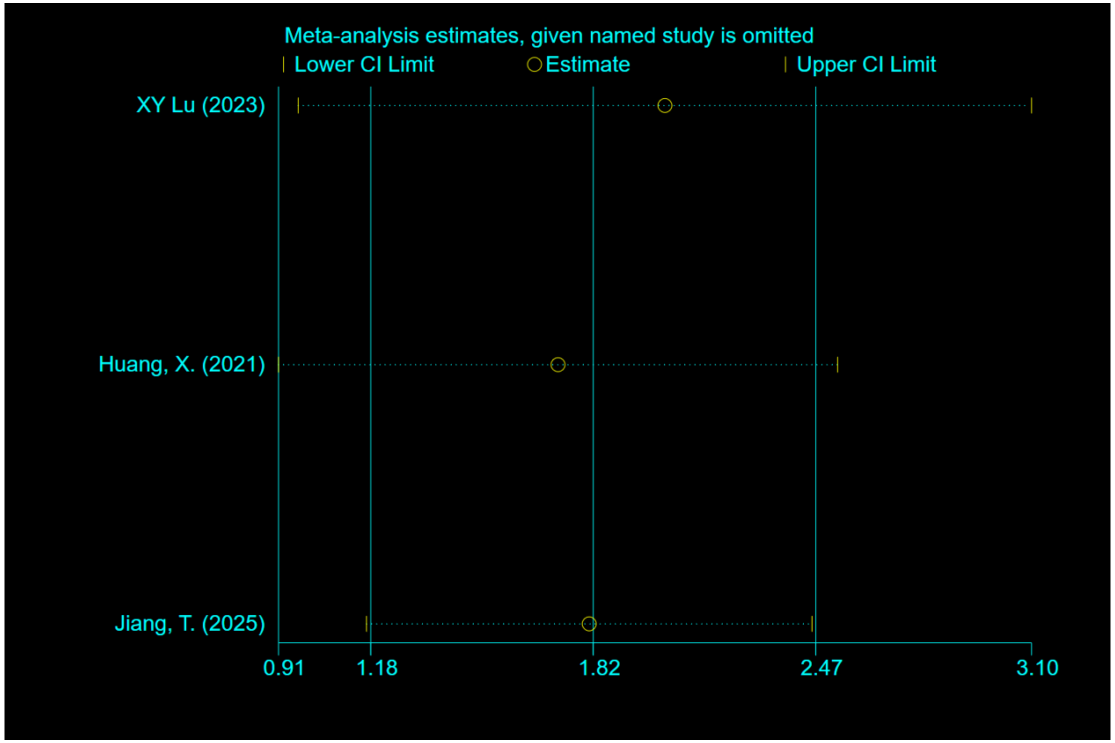

Figure S16 Sensitivity analysis of Chemotherapy cycle ≥3 (univariate meta-analysis)

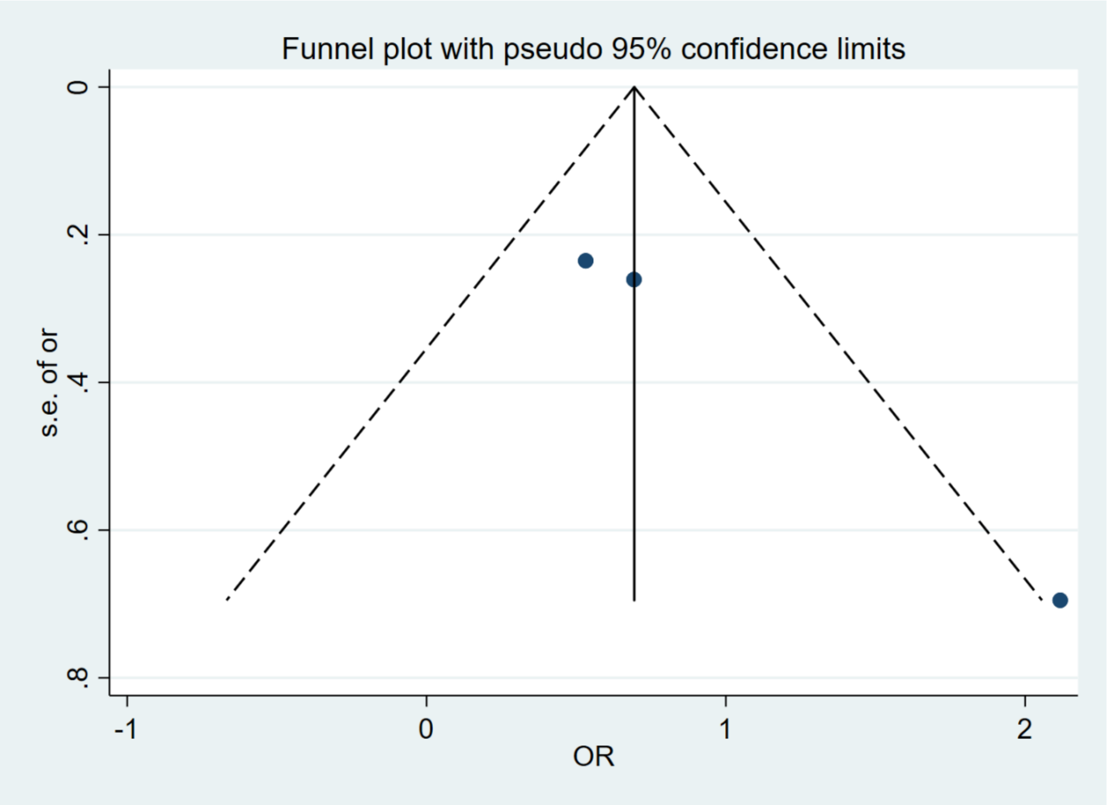

Figure S17 Funnel plot of History of Chemotherapy cycle ≥3 (multivariate meta-analysis)

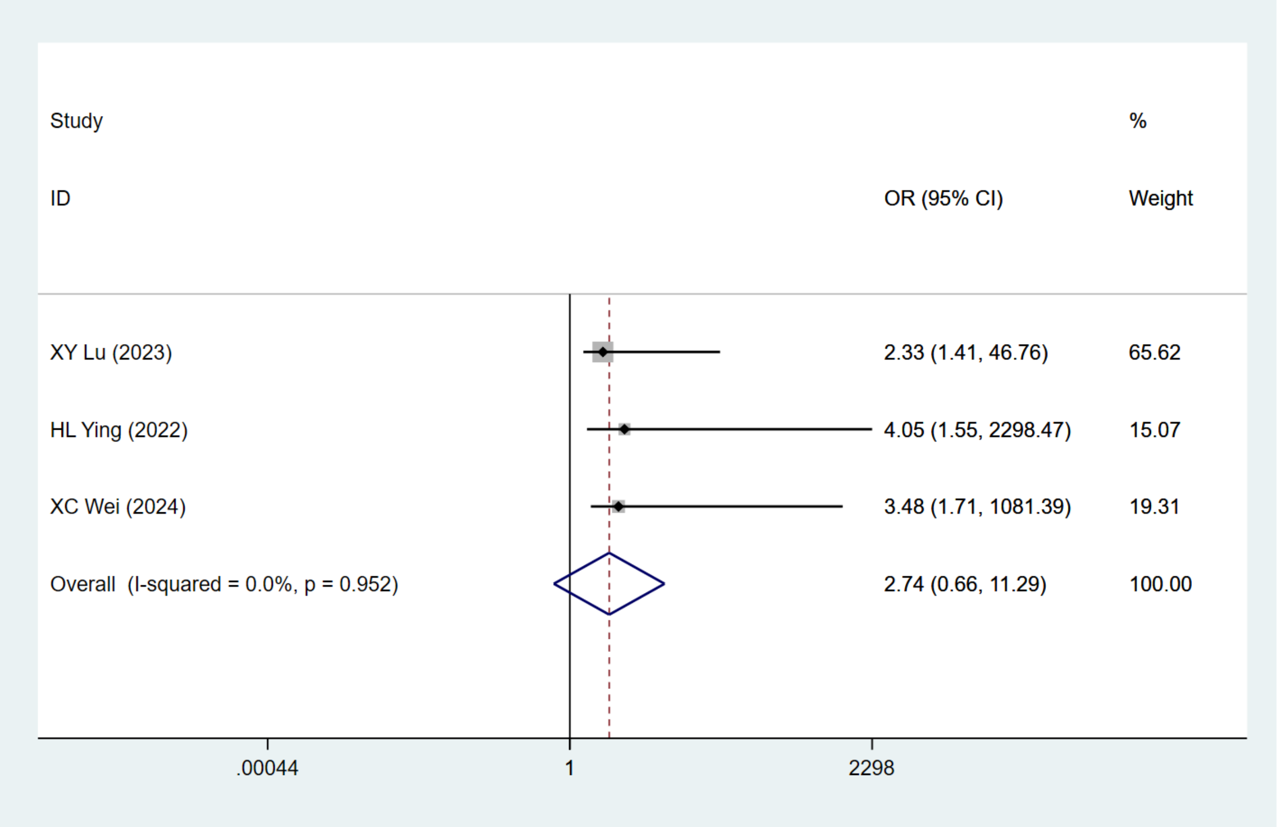

Figure S18 Forest plot of Anxiety (multivariate meta-analysis)

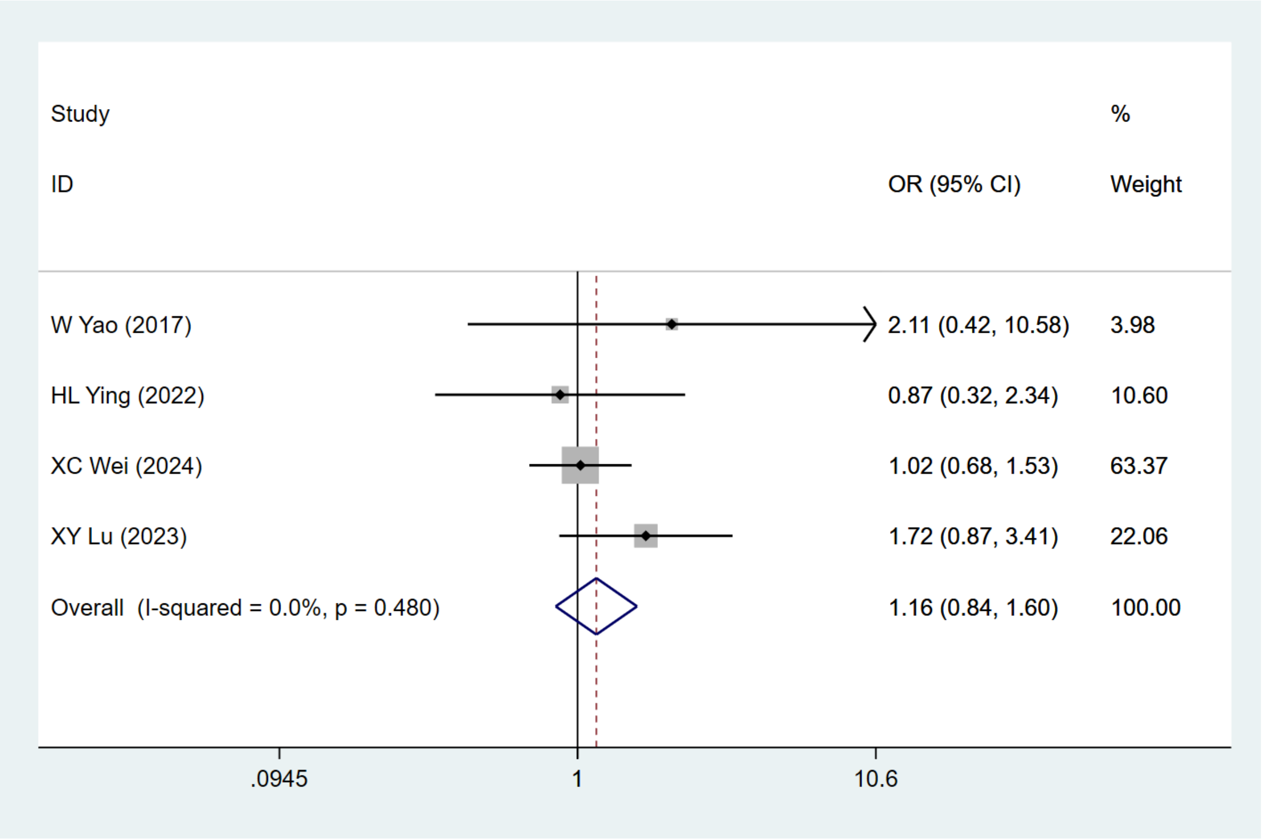

Figure S19 Forest plot of comorbidities (univariate meta-analysis)

Included Chinese literature-Attachment

AHRQ.pdf

2017 W Yao.pdf

2018 SP Quan.pdf

2022 HL Ying.pdf

2023 XY Lu.pdf

2024 XC Wei.pdf
